# Human AdV-20-42-42, a Promising Novel Adenoviral Vector for Gene Therapy and Vaccine Product Development

**DOI:** 10.1128/JVI.00387-21

**Published:** 2021-10-27

**Authors:** Mónika Z. Ballmann, Svjetlana Raus, Ruben Engelhart, Győző L. Kaján, Abdelaziz Beqqali, Patrick W. F. Hadoke, Chantal van der Zalm, Tibor Papp, Lijo John, Selina Khan, Satish Boedhoe, Katarina Danskog, Lars Frängsmyr, Jerome Custers, Wilfried A. M. Bakker, Hilde M. van der Schaar, Niklas Arnberg, Angelique A. C. Lemckert, Menzo Havenga, Andrew H. Baker

**Affiliations:** a Batavia Biosciences B.V., Leiden, The Netherlands; b Centre for Cardiovascular Sciences, University of Edinburgh, Edinburgh, United Kingdom; c Department of Clinical Microbiology, Division of Virology, Umeå University, Umeå, Sweden; d Janssen Vaccines and Prevention B.V., Leiden, The Netherlands; International Centre for Genetic Engineering and Biotechnology

**Keywords:** potent T-cell responses, cell and tissue transduction, expression vector, low seroprevalence, novel adenovirus serotype

## Abstract

Preexisting immune responses toward adenoviral vectors limit the use of a vector based on particular serotypes and its clinical applicability for gene therapy and/or vaccination. Therefore, there is a significant interest in vectorizing novel adenoviral types that have low seroprevalence in the human population. Here, we describe the discovery and vectorization of a chimeric human adenovirus, which we call HAdV-20-42-42. Full-genome sequencing revealed that this virus is closely related to human serotype 42, except for the penton base, which is derived from serotype 20. The HAdV-20-42-42 vector could be propagated stably to high titers on existing E1-complementing packaging cell lines. Receptor-binding studies revealed that the vector utilized both CAR and CD46 as receptors for cell entry. Furthermore, the HAdV-20-42-42 vector was potent in transducing human and murine cardiovascular cells and tissues, irrespective of the presence of blood coagulation factor X. *In vivo* characterizations demonstrate that when delivered intravenously (i.v.) in mice, HAdV-20-42-42 mainly targeted the lungs, liver, and spleen and triggered robust inflammatory immune responses. Finally, we demonstrate that potent T-cell responses against vector-delivered antigens could be induced upon intramuscular vaccination in mice. In summary, from the data obtained we conclude that HAdV-20-42-42 provides a valuable addition to the portfolio of adenoviral vectors available to develop efficacious products in the fields of gene therapy and vaccination.

**IMPORTANCE** Adenoviral vectors are under investigation for a broad range of therapeutic indications in diverse fields, such as oncology and gene therapy, as well as for vaccination both for human and veterinary use. A wealth of data shows that preexisting immune responses may limit the use of a vector. Particularly in the current climate of global pandemic, there is a need to expand the toolbox with novel adenoviral vectors for vaccine development. Our data demonstrate that we have successfully vectorized a novel adenovirus type candidate with low seroprevalence. The cell transduction data and antigen-specific immune responses induced *in vivo* demonstrate that this vector is highly promising for the development of gene therapy and vaccine products.

## INTRODUCTION

Adenoviral vectors have been studied for decades, as they hold great promise as tools to develop safe and effective gene therapy and vaccine products. As such, there are dozens of therapeutic applications being pursued utilizing adenoviral vectors. As it has been amply demonstrated that host immune responses limit the repeated use of a particular vector (1–4), there is a constant demand to identify new adenovirus (AdV) serotypes with low seroprevalence and alternate tropism. Thus, investigations into the *in vitro* and *in vivo* biology of less prevalent adenovirus may advance the clinical use of alternative Ad-based platforms.

To date, 104 human adenovirus (HAdV) types have been described and are subgrouped in 7 species (HAdV-A to -G), of which HAdV-D is the largest. It has been well documented that the members of the different species are associated with diverse clinical symptoms, including gastroenteritis (HAdV-F and -G), respiratory disease (HAdV-B, -C, and -E), or conjunctivitis and/or keratitis (HAdV-B and -D). HAdV-induced symptoms can be self-limiting and cleared by a host within days to weeks, but persistent infection of HAdV-C can last for months. Serotype classification is historically based on the unique neutralization profile of an adenovirus, i.e., serum specifically raised against one serotype does not cross-neutralize other adenovirus serotypes, and a hemagglutination profile. Besides the 57 acknowledged serotypes, many hybrid adenoviruses have been discovered, and as these so-called chimeras can be neutralized by parental serum, they do not qualify as distinct serotypes. Most likely such hybrids originate from homologous recombination events between two or more viruses replicating simultaneously in a host cell during coinfection (5–8).

In the present study, we describe the generation of a novel replication-incompetent vector based on a natural hybrid that we named HAdV-20-42-42. The recombinant HAdV-20-42-42 vector was used for characterization of its seroprevalence, receptor usage, and tropism. In addition, we explored the utility of the vector as a potential tool to develop gene therapy and vaccine products. The data obtained and described here warrant further studies into the utilization of the HAdV-20-42-42 vector to develop vaccines and cardiovascular intervention strategies.

## RESULTS

### Identification of HAdV-20-42-42, a natural chimera.

In order to identify possible new vector candidates, e.g., new and/or rare human adenovirus types, 281 human adenovirus strains isolated from patients in Sweden between 1978 and 2010 were screened previously (9). From these samples, the hexon, the penton base, and the polymerase genes were amplified and sequenced to allow for identification of new adenovirus types or possible recombinants. Strains with promising genotypes were propagated on A549 cells, after which the complete viral genome was sequenced using next-generation sequencing and annotated based on HAdV reference strains. Phylogenetic analyses were conducted based on the complete genome sequence and amino acid translations of the hexon, penton base, and fiber knob.

**FIG 1:**
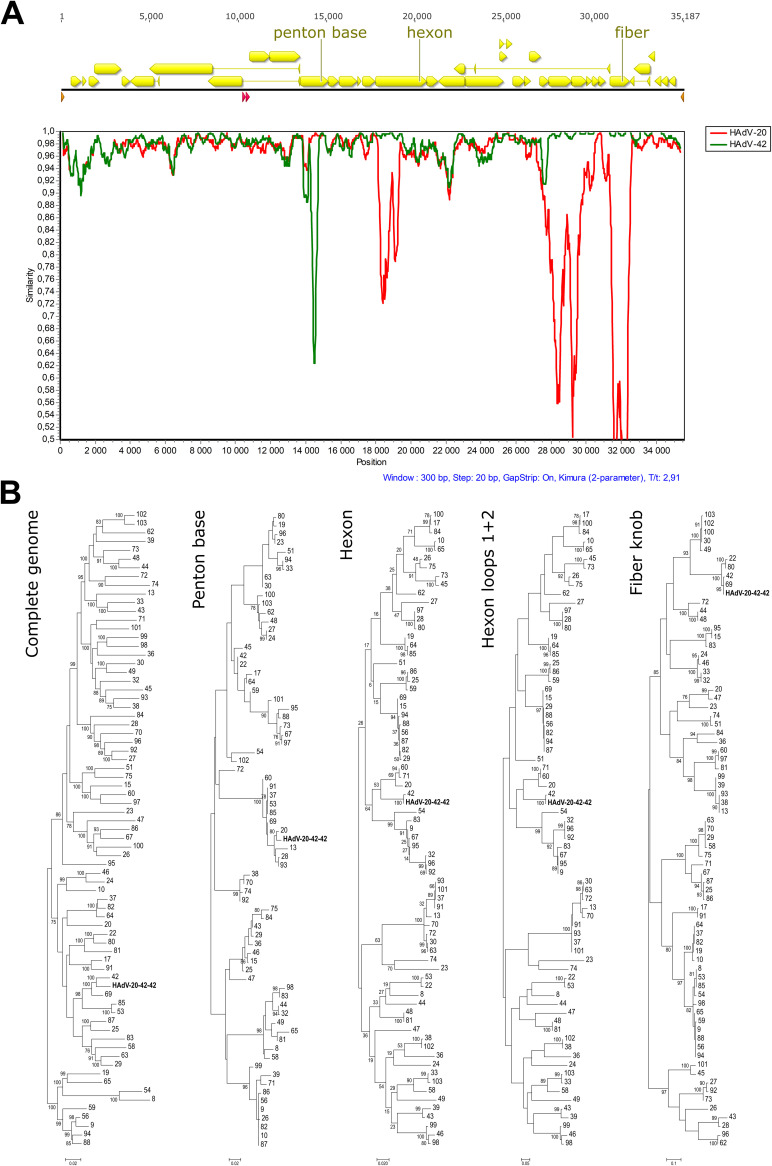
Identification of HAdV-20-42-42, a natural chimera. (A) Yellow arrows in the genome map represent protein-coding sequences, red arrows represent virus-associated RNAs, and brown arrows represent the inverted terminal repeats. In the SimPlot analysis, sequence identities to human adenovirus 20 and 42 are represented by red and green plots, respectively. (B) Phylogenetic analysis of strain 212 (Umu009) based on the complete genome sequence and derived amino acid sequences of the hexon, the penton base, the hexon loop 1, and the fiber knob. *Human mastadenovirus D* reference strains are represented by their serotype or genotype numbers.

During the screening process, strain 212 was pinpointed and analyzed further. The complete genome sequence of this strain was 35,187 bp long with a GC content of 57.0%. A typical HAdV-D genome layout was observed with 37 protein-coding sequences and two virus-associated RNAs (Fig. 1A), pointing to a recombination event of two types that resulted in a hybrid. The strain is most closely related to HAdV-42 (species *Human mastadenovirus D*) in most phylogenetic analyses except for the penton base, which has the highest sequence identity with HAdV-20 (Fig. 1B). Thus, the genomic composition of strain 212 was determined as HAdV-20-42-42 concerning the sequence of the penton base, the hexon, and the fiber knob.

### HAdV-20-42-42 shows low seroprevalence in human subjects.

**FIG 2:**
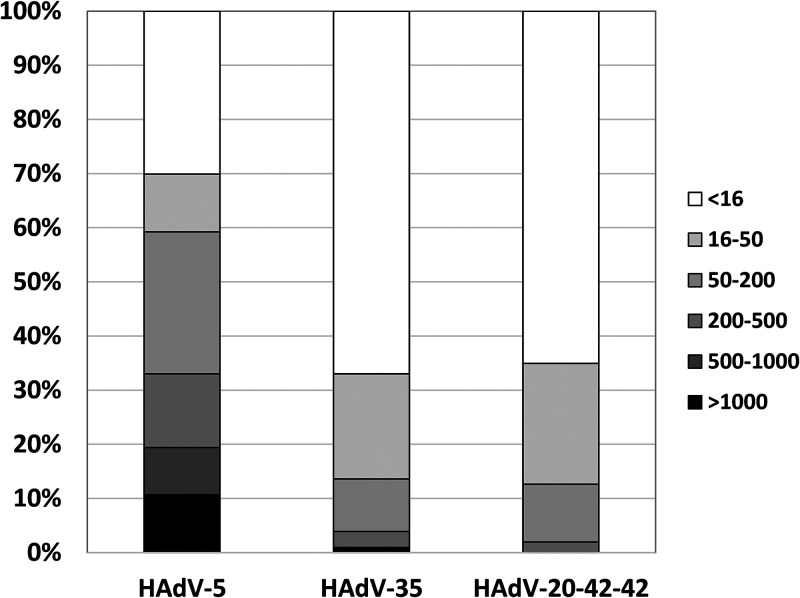
HAdV-20-42-42 shows low seroprevalence in studies with human subjects. HAdV-5, HAdV-35, and HAdV-20-42-42 seroneutralization by a cohort of healthy >50-year-old U.S. citizens (*n *=* *103 individual serum samples) is shown. The neutralization titers were arbitrarily divided into the following categories: <16 (no neutralization), 16 to 50, 50 to 200, 200 to 500, 500 to 1,000, and >1,000.

High levels of preexisting antivector humoral immunity in vaccine target populations may hamper the potential use of a novel adenoviral vector as an efficacious vaccine platform, such as that found for HAdV-5-based vectors (10–12). We investigated the levels of preexisting neutralizing antibodies against HAdV-20-42-42 using a panel of serum samples (*n *=* *103) taken from a cohort of healthy >50-year-old U.S. citizens. In line with previous findings (13), ∼60 % of the serum samples exhibited high levels of neutralizing activity (effective at dilutions of >1 : 200) against HAdV-5 (Fig. 2). For HAdV-35 and HAdV-20-42-42, on the other hand, only ∼15 % of serum samples neutralized viral activity at dilutions of >200 (Fig. 2). These data indicate that the seroprevalence of HAdV-20-42-42 in this cohort was low, with antibody levels comparable to that of the rare serotype HAdV-35 (species HAdV-B).

### Vectorization of HAdV-20-42-42.

**FIG 3:**
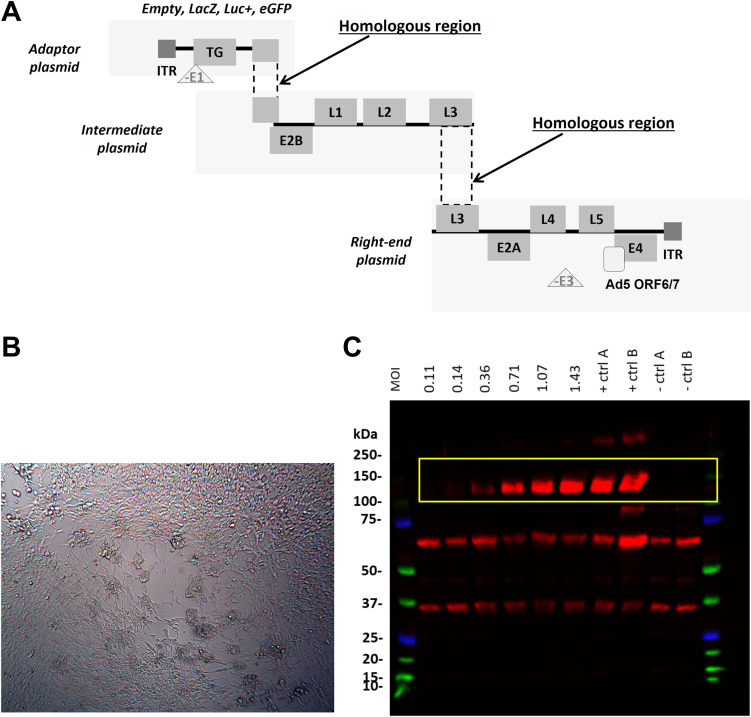
HAdV-20-42-42 vector generation and characterization. (A) Schematic representation of the replication-incompetent HAdV-20-42-42 recombinant viral vector construction strategy with a three-plasmid system (adaptor, intermediate, and right-end plasmid). Overlapping regions allowed homologous recombination events between the HAdV-20-42-42 sequences in HEK293 production cell lines. E and L represent the early and late genes of the adenoviral genome, and ITRs are the inverted terminal repeats at the 5′ and 3′ ends. (B) CPE development in HEK293 cells upon transfection of three HAdV-20-42-42 plasmids. (C) Verification of transgene (LacZ) expression. A549 cells were infected with HAdV-20-42-42-LacZ at various MOIs (MOI was calculated based on cell count at the time of cell seeding). At 3 dpi cells were lysed and subjected to Western blot analysis with an antibody against LacZ. The LacZ-specific bands are marked by a yellow rectangle. Lysates of a previous HAdV-20-42-42-LacZ infection were used as controls (+ ctrl A and B), while lysates of HAdV-20-42-42-GFP-infected cells (− ctrl A) or uninfected cells (− ctrl B) were used as negative controls.

In order to study the therapeutic applicability of HAdV-20-42-42, we first generated replication-incompetent vectors expressing reporter genes *β*-galactosidase (LacZ), luciferase (Luc), or enhanced green fluorescent protein (EGFP). To do so, engineered HAdV-20-42-42 genomic DNA sequences were cloned into three plasmids, called the adaptor plasmid, the intermediate plasmid, and the right-end plasmid, with overlapping regions to allow for homologous recombination events (Fig. 3A). To produce replication-incompetent reporter viruses, the E1 region of HAdV-20-42-42 was replaced with an expression cassette and a reporter gene in the adaptor plasmid. In the right-end plasmid, the E3 region was deleted to create capacity for insertion of larger transgenes. To enhance the growth in a standard producer cell line (i.e., HAdV-5 E1-complementing HEK293 cells), the native E4 ORF6/7 region was exchanged with that of HAdV-5 (13–15).

Reconstruction of the full-length recombinant HAdV-20-42-42 vector encoding the various reporter genes was achieved by homologous recombination via cotransfection of the three plasmids into HEK293 cells. After cotransfection, the HEK293 cells were subjected to a freeze-thaw cycle to release intracellular particles. After removal of cell debris by centrifugation, the supernatant was used for a reinfection of fresh HEK293 cells. At 3 days after reinfection of the fresh HEK293 cells, cytopathic effect (CPE) was observed (Fig. 3B) and viral progeny were successfully propagated to high titers and purified with CsCl density gradients. The reporter gene expression was detected successfully in infected cells (Fig. 3C).

### Characterization of HAdV-20-42-42 receptor usage.

**FIG 4:**
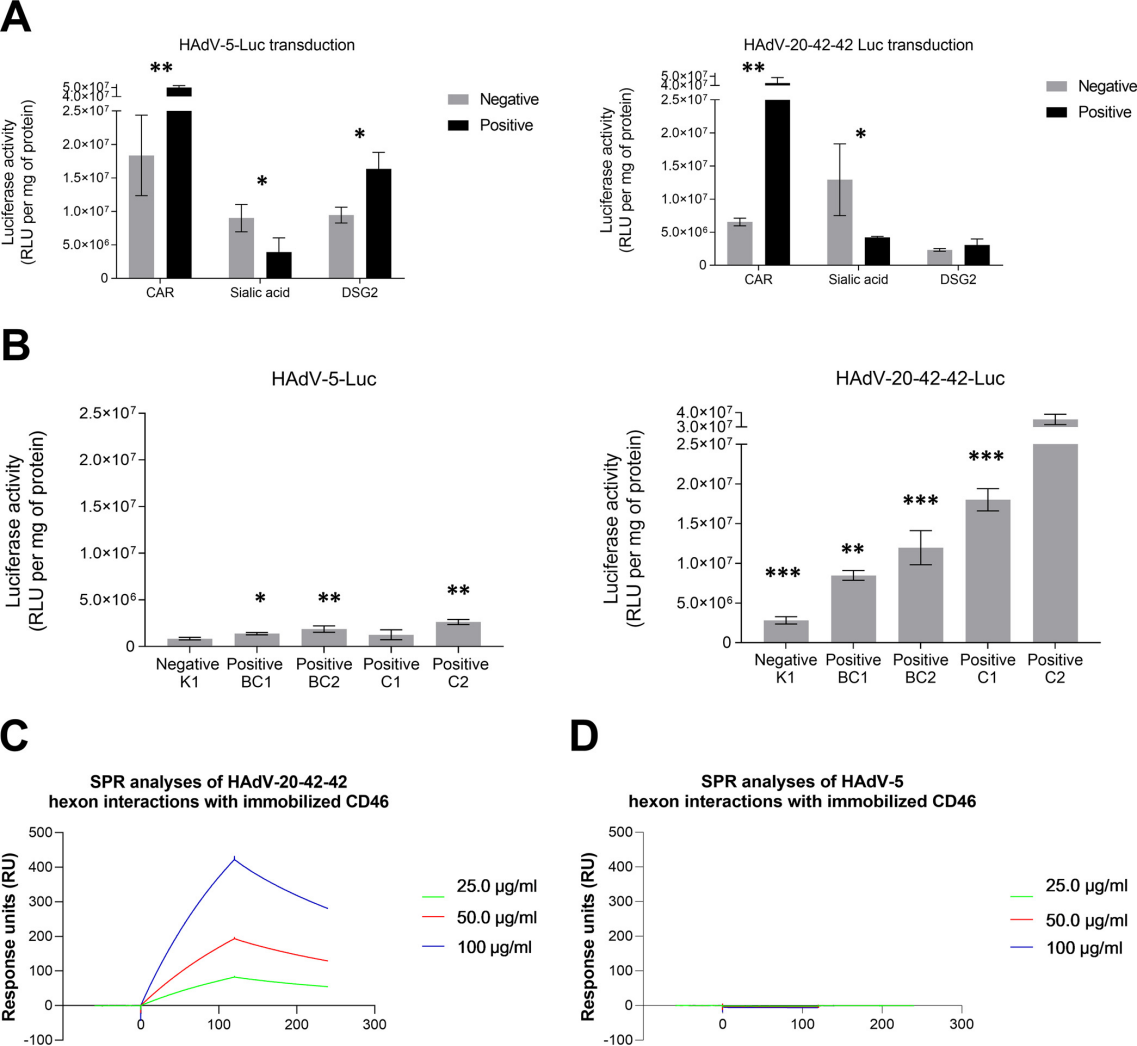
Receptor use of HAdV-20-42-42. (A) Cells expressing (positive, dark gray bars) or lacking (negative, light gray bars) CAR, sialic acid-containing glycans, or DSG2 receptors were infected with HAdV-20-42-42-Luc or HAdV-5-Luc as a control vector. (B) CHO cells lacking CD46 (K1) or expressing various CD46 isoforms (BC1, BC2, C1, or C2) were infected with HAdV-20-42-42-Luc or HAdV-5-Luc as a control vector. (A and B) One day postinfection, cells were lysed to determine intracellular luciferase activity. Luciferase activity is presented as relative light units (RLU) per milligram of protein. All results represent averaged data from experiments performed several times, with four replicates for each condition. (C and D) Surface plasmon resonance analysis of hexon (analyte) interactions with CD46 (immobilized). (C and D) HAdV-20-42-42 hexon (C) and HAdV-5 (D) interactions with CD46. Error bars are presented as standard errors of the means (SEM). A *P* value of <0.05 by two-sample, two-tailed Student’s *t* test was considered statistically significant (*, *P* < 0.05; **, *P* < 0.01). Statistical significance was calculated for positive versus negative cells (A) or compared to the negative cell line K1 (B).

Well-studied adenovirus gene therapy or vaccination vectors (e.g., HAdV-5, HAdV-26, and HAdV-35) bind their fibers to CD46, coxsackie and adenovirus receptor (CAR), or desmoglein 2 (DSG2) as high-affinity receptors, while their penton bases interact with αν-integrins as coreceptors for internalization (16). HAdV-20-42-42 clusters to a small group of HAdV-Ds, which have been previously shown to utilize subunits of sialic acid as primary receptors for cellular attachment and/or entry. To investigate the receptor usage, we determined the transduction capacity of HAdV-20-42-42-Luc in various cell lines, expressing or lacking CAR, sialic acid-containing glycans, DSG2, or CD46 isoforms, by measuring the luciferase levels after infection. Similar to HAdV-5, HAdV-20-42-42 efficiently transduced CHO-CAR cells but was also able to transduce Chinese hamster ovary (CHO) cells lacking the CAR receptor, albeit at a much lower efficiency (Fig. 4A), suggesting that it also utilizes other receptors.

Although being exploited by other HAdV-D members for infection, the presence of sialic acid on cells did not increase the transduction capacity of HAdV-20-42-42. Levels of DSG2 in TC1-DSG2 monocytes also did not affect the luciferase expression of HAdV-20-42-42 (Fig. 4A). Next, the transduction efficiency was evaluated in CHO cells expressing various isoforms of CD46 (Fig. 4B). CHO-K1 cells, which lack CD46, were poorly transduced by HAdV-20-42-42. While HAdV-5 transduced all cell lines weakly and only marginally more efficiently than the control cell line CHO-K1, HAdV-20-42-42 transduced CHO-K1 cells expressing all CD46 isoforms more efficiently than the control cell line. Cells expressing the C2 isoform were transduced more efficiently. We recently demonstrated that HAdV-26 and -56 bind to CD46 via the hexon protein (17). Surface plasmon resonance data demonstrated that the hexon protein of HAdV-20-42-42, which belongs to the same species (D) as HAdV-26 and HAdV-5, also bound to CD46, but the hexon of HAdV-5 did not (Fig. 4C and D, respectively). The affinity and interaction half-life were determined to be 13.5 μM and 187 s, respectively (not shown). Together, these findings indicate that the novel adenoviral vector HAdV-20-42-42 is able to bind to both CAR and CD46 receptors, primarily the C2 isoform, and that the interaction with CD46 is mediated by the hexon protein. As these receptors are present in many cell types, this warrants a broad use of the novel adenoviral vector HAdV-20-42-42 in gene therapy.

### HAdV-20-42-42 vector interactions with serum and coagulation factors.

**FIG 5:**
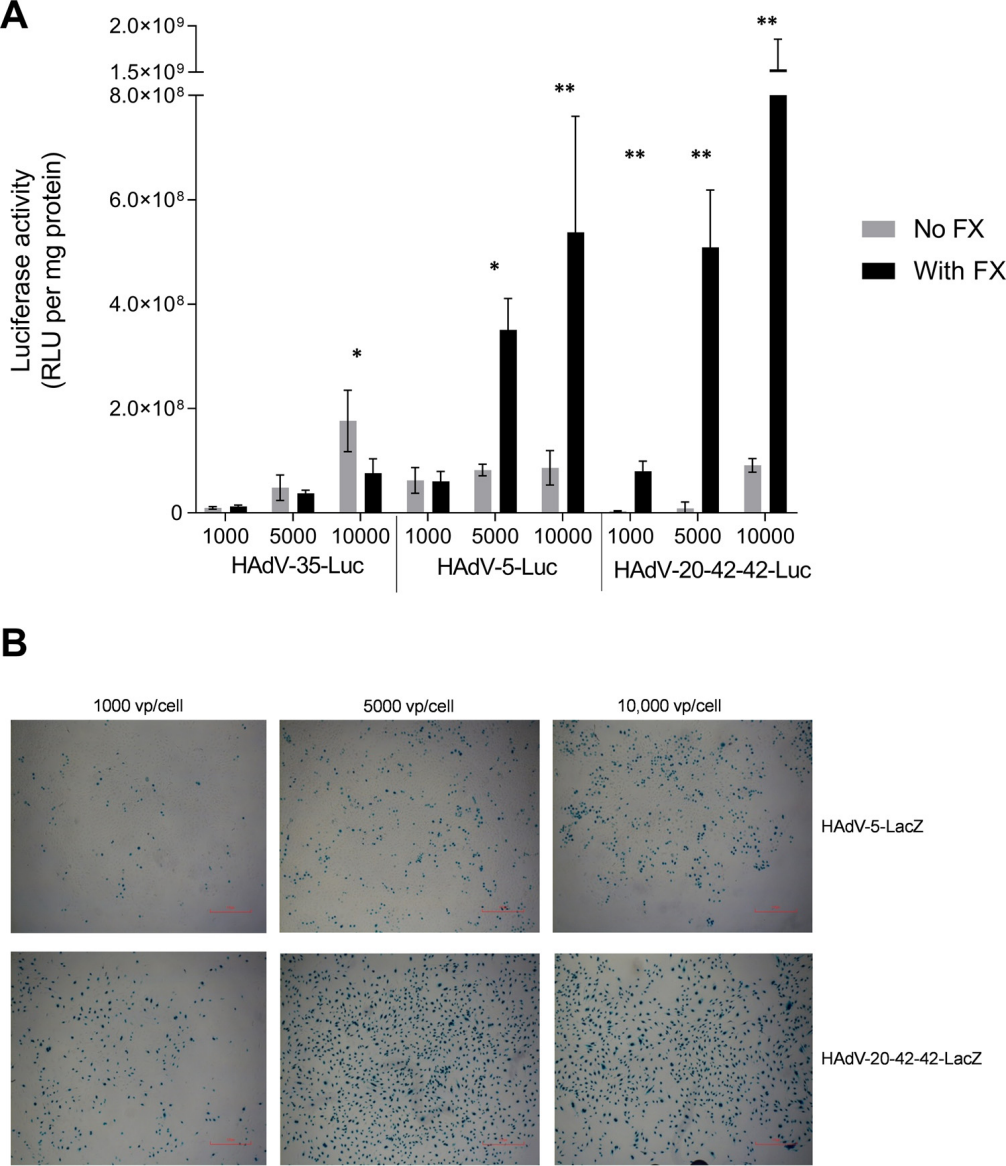
Transduction efficiency of HAdV-20-42-42 in vascular cells. HAdV-20-42-42-Luc and -LacZ vectors were tested for transduction capacity in HSVEC (human saphenous vein endothelial cells). (A) HSVEC were infected with HAdV-20-42-42-Luc or the control vectors HAdV-35-Luc and HAdV-5-Luc at various doses (1,000, 5,000, or 10,000 VP/cell) for 3 h. Where indicated (dark gray bars), the vectors were incubated for 30 min at 37°C with a physiological concentration of 10 μg/ml blood coagulation factor FX prior to addition to the cells. After 2 days, cells were lysed to measure intracellular luciferase activity, which is presented as relative light units (RLU) per milligram of protein. Bars represent the means plus standard errors of the means (SEM) (error bars) for quadruplicate values. A *P* value of <0.05 by two-sample, two-tailed Student’s *t* test was considered statistically significant (*, *P* < 0.05; **, *P* < 0.01). All results represent averaged data from several experiments, with four replicates for each condition. (B) HSVEC were infected with various doses of HAdV-20-42-42-LacZ or HAdV-5-LacZ in the presence of FX. After 2 days, cells were stained for LacZ expression.

The binding of many HAdV types to human coagulation blood factor X (FX) significantly affects the transduction *in vitro* and the tropism *in vivo* following intravenous (i.v.) administration (10, 18, 19). We investigated whether the presence of FX impacts the transduction efficiency of HAdV-20-42-42. Physiological concentrations of FX were incubated with cells prior to the addition of HAdV-5, -35, or HAdV-20-42-42 luciferase vectors, and intracellular luciferase levels were measured 2 days after transduction. While HAdV-35 transduction was only marginally affected by the addition of FX, the luciferase levels of HAdV-5 and, in particular, of HAdV-20-42-42 were substantially increased in the presence of FX (Fig. 5A). These data show a notably higher transduction potential of HAdV-20-42-42 over HAdV-5 and HAdV-35 in human saphenous vein endothelial cells (HSVEC) in the presence of FX. HSVEC were infected with HAdV-20-42-42-LacZ and HAdV-5-LacZ as a control. At all doses tested, the percentage of LacZ-positive cells was higher for HAdV-20-42-42 than HAdV-5 (Fig. 5B). These data show that HAdV-20-42-42 was capable of efficiently transducing vascular cells in the presence of FX.

### HAdV-20-42-42 biodistribution following systemic delivery *in vivo*.

**FIG 6:**
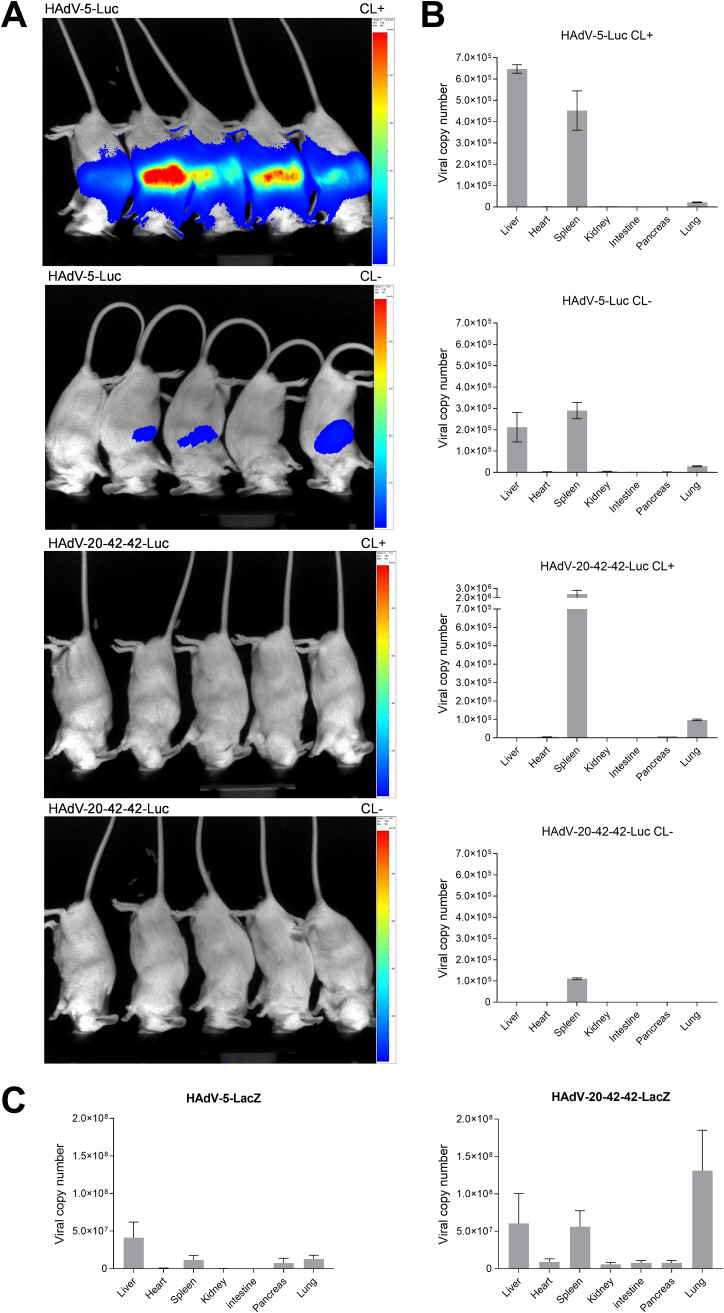
Biodistribution profile of HAdV-20-42-42 shows mainly spleen tropism. Mice were pretreated with clodronate (CL+) or untreated (CL−) and injected intravenously with HAdV-5-Luc or HAdV-20-42-42-Luc vectors. (A) At 48 h after virus delivery, luciferin was injected into the mice and luciferase activity was imaged with the IVIS Spectrum method, which ranged from low activity (shown in blue) to high activity (shown in red) levels. (B) After imaging, animals were sacrificed and organs were collected to determine adenoviral genome copy numbers with quantitative PCR (qPCR). Data represent viral copy number per 100 ng of total DNA. Bars represent the means plus standard errors of the means (SEM). (C) Biodistribution profile 1 h after injection as determined by qPCR. Data represent viral copy number per 100 ng of total DNA. Bars represent the means plus standard errors of the means (SEM).

To characterize the HAdV-20-42-42 vectors *in vivo* following intravenous administration, we evaluated the biodistribution patterns of HAdV-20-42-42-Luc using a previously described mouse model (20, 21). Animals inoculated with vehicle (phosphate-buffered saline [PBS]) or HAdV-5-Luc were included as negative- and positive-control groups, respectively. Pretreatment with clodronate liposomes (CL+) was performed on half of the animals in order to deplete macrophages, thereby reducing possible sequestration of the adenovirus vector to the liver and allowing a more efficient evaluation of the biodistribution at the whole organism level. Two days after vector administration, the animals were imaged to visualize luciferase levels (Fig. 6A). Subsequently, the mice were sacrificed and several organs were collected for adenoviral DNA quantification (Fig. 6B).

In the control group of animals without CL pretreatment, HAdV-5-Luc was mainly distributed in liver and spleen at levels of ∼2.5 × 10^5^ genome copy numbers per 100 ng total DNA, while in the group pretreated with CL the liver and spleen distribution was higher, closer to ∼5 × 10^5^ genome copy numbers (Fig. 6). HAdV-20-42-42, on the other hand, appeared to have only a spleen tropism, since luciferase activity was not detected in other organs. As expected, the total DNA copy number was significantly higher when CL was added (∼2.5 × 10^6^) compared to that of the group without CL pretreatment (1 × 10^5^).

In order to address the short-term organ biodistribution after administration, mice were injected intravenously with PBS, HAdV-5-LacZ, and HAdV-20-42-42-LacZ and sacrificed 1 h post-i.v. delivery. The biodistribution profile of HAdV-20-42-42-LacZ was similar to that of HAdV-5-LacZ, with sequestration mainly observed in liver, spleen, and lungs (Fig. 6C).

### HAdV-20-42-42 as a candidate vaccine vector.

**FIG 7:**
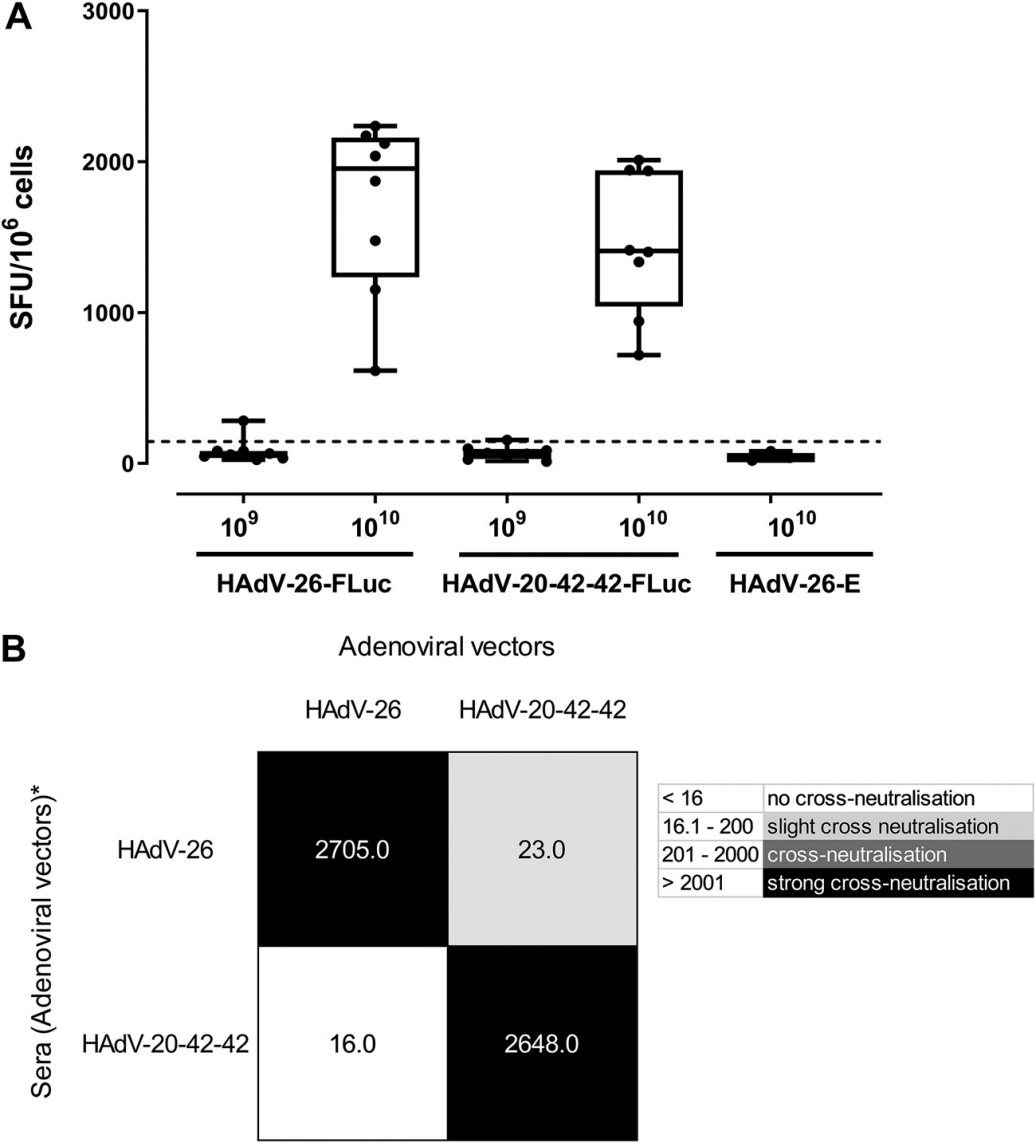
Candidate vector HAdV-20-42-42 elicits strong immune response in mice. (A) Cellular immune response in mice. BALB/c mice were immunized by intramuscular injection with HAdV-26-Luc, HAdV-20-42-42-Luc, or HAdV-26-E, which lacks a transgene. Two vector doses, i.e., 10^9^ and 10^10^ viral particles per mouse, were administered, and animals were sacrificed 2 weeks postimmunization and sampled for serum and splenocytes. Cellular immune responses against the vector-encoded antigen was evaluated by Luc-specific-IFN-γ ELISPOT assay. To this end, splenocytes were stimulated overnight with a 15mer overlapping Luc peptide pool. The antigen-specific immune responses were determined by measuring the relative number of IFN-γ-secreting cells, shown as spot-forming units (SFU) per million cells. Each dot represents a mouse, the bar indicates the geometric mean, and the dotted line is the 95th percentile based on the medium control samples. (B) Cross-neutralization between HAdV-20-42-42 and HAdV-26. Mouse antisera against HAdV-20-42-42 and HAdV-26 were cross-tested against both vectors in an adenovirus neutralization assay. Starting from a 1:16 dilution, the sera were 2-fold serially diluted, premixed with the adenoviral vectors expressing luciferase, and subsequently incubated overnight with A549 cells at an MOI of 500 virus particles. Luciferase activity levels in infected cell lysates measured 24 h postinfection represented vector infection efficiencies. The neutralization titers were arbitrarily divided into the categories shown in the legend on the right.

The potential of HAdV-20-42-42 as a vaccine vector candidate was assessed for its ability to induce cellular immune responses against a model antigen (Luciferase [Luc]) in BALB/c mice after intramuscular immunization. The vector was compared side-by-side with a benchmark vector based on HAdV-26, which has undergone clinical trials for HIV, Ebola, and, recently, for SARS-CoV-2 (22–25). Mice were immunized intramuscularly with two different doses of E1- and E3-deleted HAdV-26 vector expressing luciferase (HAdV-26-Luc) or HAdV-20-42-42-Luc. Mice were sacrificed and sampled for serum and splenocytes 2 weeks after the prime immunization. Cellular immune responses against the vector-encoded antigen were evaluated by Luc-specific gamma interferon (IFN-γ) enzyme-linked immunosorbent spot (ELISPOT) assay. To this end, splenocytes sampled from immunized mice were stimulated overnight with a 15mer overlapping Luc peptide pool. The antigen-specific immune responses were determined by measuring the relative number of IFN-γ-secreting cells (Fig. 7A). As expected, no Luc-specific responses were detected against the empty adenovectors lacking luciferase (HAdV-26-E). Furthermore, the results show that the cellular immune responses induced by HAdV-20-42-42 were comparable to the response seen for HAdV-26 at the highest immunization dose (10^10^ VP).

For their potential utility as new adenoviral vaccine vectors, the novel HAdV-20-42-42 adenoviral vector would preferably be serologically distinct from existing adenoviral vectors currently in development as vaccine vectors, such as HAdV-26. Therefore, cross-neutralization tests were performed between the novel HAdV-20-42-42 adenoviral vector and HAdV-26. To this end, mouse antisera raised against these vectors during the immunization study described above were cross-tested against both vectors in an adenovirus neutralization assay. The adenovirus neutralization assay was carried out as described previously (26). Briefly, starting from a 1:16 dilution, the sera were 2-fold serially diluted, premixed with the adenoviral vectors expressing luciferase (Luc), and subsequently incubated overnight with A549 cells at a multiplicity of infection (MOI) of 500. Luciferase activity levels in infected cell lysates measured 24 h postinfection represented vector infection efficiencies. Neutralization titers against a given vector were defined as the highest serum dilution capable of giving a 90% reduction of vector infection efficiency. The neutralization titers were arbitrarily divided into the following categories: <16 (no neutralization), 16.1 to 200 (low cross-neutralization), 201 to 2,000 (cross-neutralization), and >2,001 (strong cross-neutralization).

The results show no major cross-neutralization between the vectors tested (Fig. 7B), but a low, one-way cross-neutralization was observed with HAdV-26 antiserum displaying a neutralization titer against HAdV-20-42-42 of 23. Thus, the new adenoviral vector HAdV-20-42-42 displayed low, if any, cross-neutralization with the human adenoviral vector HAdV-26.

**FIG 8:**
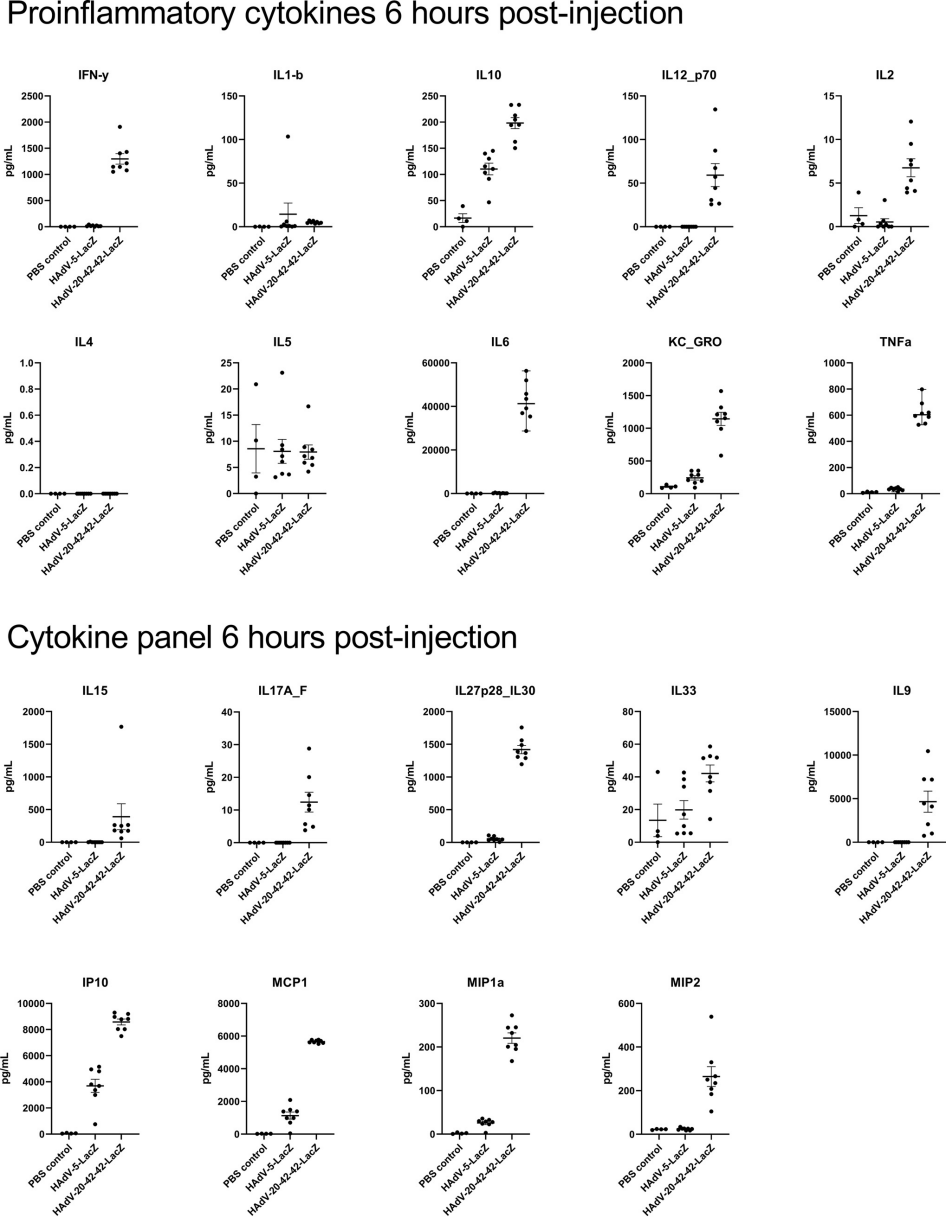
Cytokine profiles upon i.v. delivery of vectors in mice. Cytokines were measured in blood serum samples from mice at 6 h postinjection of PBS (control), HAdV-5-LacZ, and HAdV-20-42-42-LacZ (1 × 10^11^ VP).

Previous reports have demonstrated a potent inflammatory immune response against Ad vectors in the hours following i.v. administration. We investigated the levels of cytokines induced following i.v. delivery of PBS, HAdV-20-42-42-LacZ, or HAdV-5-LacZ in mice. Serum samples were collected at 6 h post-i.v. administration and were tested against a mouse multiplex cytokine/chemokine panel. HAdV-20-42-42 induced higher levels of proinflammatory mediators, and, in general, these were elevated above the levels observed in HAdV-5 or PBS inoculation in 14 out of the 19 cytokine/chemokines measured (Fig. 8). Specifically, IFN-γ, interleukin-10 (IL-10), IL12_p70, IL-6, KC_GRO, tumor necrosis factor alpha (TNF-α), IL-15, IL17A_F, IL-27 p28_IL30, IL-9, IP10, MCP1, MIP1a, and MIP2 were robustly upregulated in HAdV-20-42-42-injected animals.

Taken together, these data indicate that HAdV-20-42-42 triggers a robust inflammatory and cellular immune response *in vivo* following i.v. delivery.

## DISCUSSION

We present the first report on the generation of a replication-incompetent HAdV-20-42-42 vector and present data on initial *in vitro* and *in vivo* characterization. Our serum neutralization studies using wild-type (WT) HAdV-20-42-42 virus demonstrated that this virus displayed low seroprevalence in a random set of sera derived from healthy U.S. subjects. Low seroprevalence has been an important criterion to select a virus for vector development, as it has been amply demonstrated that the transduction capability of the vector can be hampered in the presence of a high titer of neutralizing antibodies (27).

Based on these initial data, we set out to generate a vector system based on HAdV-20-42-42. We created a flexible three-plasmid system to support HAdV-20-42-42 vector generation, allowing for the convenient insertion of transgenes into a multiple cloning site. The wild-type HAdV-20-42-42 E4ORF6 region was replaced with that of Ad5, a technique that we previously adopted for the generation of other species HAdV-D viruses, including HAdV-26, HAdV-48, HAdV-49, and HAdV-56 vectors. This replacement allowed for their successful production in Ad5 E1-complementing cells, such as HEK293 and PER.C6 (13, 28, 29), and enabled us to manufacture high-quality, high-titer HAdV-20-42-42 vectors carrying diverse inserts, which paves the road for large-scale clinical production.

Previous studies have demonstrated that HAdV-D strains can employ CAR, CD46, sialic acid-containing glycans, and αv-integrins as entry receptors (16). In accordance with this, we demonstrate here that HAdV-20-42-42 utilizes CAR and CD46 as receptors. Strikingly, HAdV-20-42-42, but not HAdV-5, binds to CD46 via the hexon protein, which is far more abundant on the virus capsid than the fiber (i.e., 240 trimers versus 12 trimers, respectively), which can make hexon–CD46-interacting vectors novel, useful features compared to other adenovirus-based vectors that bind to CAR or CD46 through fibers. Similar to HAdV-26 and HAdV-48, this virus interacts with blood coagulation factors (10). Together with data demonstrating the high transduction efficiency of cardiovascular cells *in vitro*, these findings suggest that this vector is well suited to developing cardiovascular gene therapy approaches, although further *in vivo* studies are required to develop this concept in more detail, since the route of delivery defines tropism, delivery, and inflammatory responses.

In addition, the data obtained from our vaccination experiments suggest that this vector is capable of eliciting potent insert-specific T-cell responses at levels similar to those of an HAdV-26 vector. The HAdV-26 vector platform has recently been successfully used to develop a potent and safe vaccine against Ebola, and this vaccine has been approved for human use by European regulatory authorities. In addition, this vector is being tested for the development of preventive vaccines against HIV, respiratory syncytial virus, and, more recently, SARS-CoV-2 (22, 23, 30). In preclinical tests, HAdV-26 used alone was demonstrated to induce robust humoral and cellular immunity, plus it has been well tolerated in humans while eliciting target-specific immunity in phase I to III trials. We were interested in testing cross-neutralization between both D viruses to assess whether subsequent use of these vectors would be a possibility. Our data demonstrate that serum from HAdV-26 does not neutralize our HAdV-20-42-42 and vice versa.

The induction of the potent adaptive immunity required for successful vaccination vectors is dependent on sufficient activation of the innate immune system. Vaccine vectors benefit from complement activation and the production and release of cytokines and chemokines, including IL-6, TNF-α, IFN-γ, MCP1, IP10, and MIP1, which are major players in the induction of antiviral immune responses. HAdV-20-42-42 induced all the aforementioned mediators. These same factors may translate to AdV-associated toxicity, making HAdV-20-42-42 unsuitable for intravascular delivery, however. Direct translation of innate responses obtained in mice, which lack the CD46 receptor, to the human situation needs caution. It has been shown that AdV vector-induced innate cytokine responses are triggered largely by receptor binding (31). Nonhuman primates may be a better model to gain insight in the induced innate immune profiles of HAdV-20-42-42 compared to mice, as the latter lack the functional cellular receptor CD46. Therefore, impact assessment of the cytokine profiles on generation of adaptive immune responses as well as safety require further studies in additional species. Regarding seroprevalence, our study was limited in its geographical distribution and age. Further studies are required to assess this in wider cohorts to fully ascertain seroprevalence. To summarize, our studies into the HAdV-20-42-42 chimera demonstrate that we have identified a promising novel adenoviral vector to pursue both gene therapy and vaccine applications and further preclinical and clinical studies utilizing this vector are warranted.

## MATERIALS AND METHODS

### Origin and sequencing of HAdV-20-42-42.

Strain 212 was isolated in the Skåne University Hospital, Lund, Sweden, in 1978 (9). To obtain the complete genome sequence, it was propagated on human alveolar epithelial cells (A549), and the intracellular viral DNA was purified from infected cells (32). The genome was sequenced using Ion Torrent next-generation sequencing at the Uppsala Genome Center of the National Genomics Infrastructure (SciLifeLab, Uppsala, Sweden). The resulting reads were normalized to a 60× coverage using BBNorm from the BBTools suite. The normalized reads were assembled *de novo* using Mira v4.9.5_2 (33), and the original sequence reads were mapped to the resulting consensus sequence using the Geneious mapper at the highest sensitivity with five iterations in Geneious 9.1.8 (34). After mapping the sequence reads to the *de novo* assembly, the final read coverage minimum was 51, the mean was 1,048.4, and the read coverage’s standard deviation was 274.5. The new consensus sequence was annotated based on HAdV reference strain genome annotations, using the Annotate & Predict function of Geneious.

### Phylogenetic analysis.

Phylogenetic analyses were conducted based on the complete genome sequence and derived amino acid sequences of the entire hexon and penton base and also on hexon loop 1 (delimited according to Yuan et al. [35]) and the fiber knob. For phylogenetic tree inference, multiple alignments were conducted using MAFFT (36), and phylogenetic calculations were performed using RAxML-NG v0.9.0 (37) based on alignments edited in trimAl v1.2 (38). Evolutionary model selection was performed using ModelTest-NG v0.1.5 (39). The robustness of the trees was determined with a nonparametric bootstrap calculation using 1,000 repeats. Phylogenetic trees were visualized using MEGA 7 (40), trees were rooted on the midpoint, and bootstrap values are given as percentages if they reached 75%. Recombination events were analyzed using SimPlot v3.5.1 (41).

### HAdV seroneutralization.

Serological inhibition of HAdV-20-42-42, HAdV-35, and HAdV-5 transduction was evaluated over a collection of 103 serum samples from a cohort of healthy >50-year-old U.S. citizens. Seroneutralization assays were performed using the protocol described in Sprangers et al. (26). Briefly, serum samples were heat inactivated at 56°C for 60 min, and then 2-fold dilutions were performed in a 96-well tissue culture plate. The dilutions covered a range from 1/16 to 1/4,096 in an end volume of 50 μl Dulbecco's modified Eagle's medium (DMEM). Negative controls consisted of DMEM alone. After addition of 50 μl virus solution (1 × 10^8^ VP ml^−1^) to each well, a cell suspension (10^4^ A549 cells) was added to the well to a final volume of 200 μl. Following 24 h of incubation, the luciferase activity in the cells was measured using a Steady-Glo luciferase reagent system (Promega). The neutralization titers were defined as the maximum serum dilution that neutralized 90 % of luciferase activity.

### Cell lines.

HEK293 (human embryonic kidney cells; ATCC CRL-1573) were grown in DMEM supplemented with 10 % fetal bovine serum (FBS; Gibco, UK). A549 (human lung epithelial carcinoma; ATCC CCL-185) cells were grown in RPMI 1640, supplemented with 10 % FBS, 1 % penicillin-streptomycin (P/S), 1 % l-glutamine (Gibco), and 1 % Na-Pyr (Sigma, UK). Chinese hamster ovary (CHO) cells (42), CHO-CAR (42), and various CHO-CD46 cells (43) were grown as described before. TC1-DSG2 cells, a kind gift of A. Lieber (University of Washington, Seattle, WA, USA), were grown in RPMI supplemented with 10% FBS and 20 mM HEPES.

### Construction of replication-incompetent recombinant HAdV-20-42-42 vectors.

The wild-type HAdV-20-42-42 virus was plaque purified, propagated on HEK293 cells, and purified by cesium chloride (CsCl) density gradient centrifugation. From the purified virus material, full genomic DNA was isolated that served as starting material for the construction of the HAdV-20-42-42 plasmids.

### HAdV-20-42-42 cloning system.

The HAdV-20-42-42 vector construction strategy was based on a three-plasmid system with sufficient homology between each of the plasmids to enable homologous recombination *in vitro* following the cotransfection in HAdV-5 E1-complementing HEK293 cells. Adapter plasmids that contain the left end of the HAdV-20-42-42 genome with E1 deletions and include either luciferase, EGFP, or LacZ reporter genes were generated first. Briefly, the adapter plasmid pAdApt20-42-42 (nucleotide [nt] 1 to 461 of WT HAdV-20-42-42) contained the left inverted terminal repeat (ITR) and included an expression cassette consisting of a cytomegalovirus promoter followed by a multiple cloning site, encompassing Luc, GFP, or LacZ, and the simian virus 40 poly(A) signal. This plasmid also contained the wild-type HAdV-20-42-42 nt 3361 to 5908 to allow homologous recombination in HEK293 cells, with the intermediate plasmid carrying the HAdV-20-42-42 genome from IVa2 to L3 genes (nt 2088 to 18494). The right-end plasmid contained the HAdV-20-42-42 genome from the L3 gene to the right ITR (nt 15373 to 35187) and had a deletion for the E3 region, while the HAdV-5 E4-ORF6 replaced the HAdV-20-42-42 E4-ORF6. The E3 region was deleted by PCR and standard cloning techniques, exploiting a natural AscI site in the viral genome. To replace the native ORF6/7 with the homologue region of HAdV-5, three fragments were amplified by PCR. Two were designed to cover the region upstream and downstream from ORF6/7 to be replaced. A third PCR was performed to obtain the HAdV-5 ORF6/7 (HAdV-5 GenBank accession no. M73260, nt 32963 to 34077) and partly overlapping the other two PCR products. These three PCR products were then subjected to fusion PCR and cloned into the plasmid backbone to obtain the final right-end plasmid.

### Generation and production of HAdV-20-42-42-based adenoviral vectors.

Adenoviral vectors HAdV-20-42-42-LacZ, HAdV-20-42-42-Luc, and HAdV-20-42-42-GFP were generated by cotransfection of E1-complementing HEK293 cells with the adaptor plasmid, intermediate plasmid, and right-end plasmid. Prior to transfection into HEK293 cells, the three plasmids were digested with PacI to release the respective adenoviral vector genome fragments. The transfections were performed using Lipofectamine transfection reagent (Invitrogen, Carlsbad, CA) according to the manufacturer’s instructions. After harvesting of the viral rescue transfections, the viruses were further amplified by several successive infection rounds on HEK293 cell cultures. The viruses were purified from crude viral harvests using a two-step cesium chloride (CsCl) density gradient ultracentrifugation procedure as described before (14). Viral particle (VP) titers were measured by a spectrophotometry-based procedure described previously (44).

### Viral transduction assays.

The transduction assay was performed on 96-well tissue culture plates (Costar). HSVEC were seeded at 1 × 10^4^ cells/well. The following day monolayers were washed with PBS, and viral vectors, encoding the luciferase or *β*-galactosidase gene, were added at the indicated VP/cell concentrations in their corresponding media without serum. In the experiments in which blood coagulation factor FX was used, it was preincubated with the vector for 30 min at 37°C prior to addition to the cells. The FX coagulation factor was purchased from Cambridge Bioscience and used at a physiological concentration of 10 μg/ml. After 3 h of incubation at 37°C, the cells were washed and fresh medium supplemented with 10 % FBS was added, after which the cells were incubated for 48 h. For the readout of *β*-galactosidase gene expression, staining of the cells was performed using the Galacto-Light Plus assay kit (Thermo Fisher Scientific). For the readout of luciferase activity, the plates were washed with PBS and the cells were lysed with 1× reporter lysis buffer (100 μl per well; Promega). Following a freeze-thaw cycle, luciferase (Luciferase assay system; Promega) measurements were performed with 20 μl of lysed cells in white opaque plates (Greiner BioOne) by following the manufacturer’s instructions. The bicinchoninic acid protein quantitation assays (Thermo Fisher Scientific) were performed with 20 μl lysed cells. The results were recorded in a Victor X multilabel plate reader (Perkin-Elmer). The transduction level was expressed as luminescence relative light units per milligram of protein per well (RLU/mg). All of the assays were performed with four replicates of samples.

### Receptor usage assays.

Studies of receptor usage were performed using CHO cells, which were expressing (positive) or lacking (negative) receptors of interest. The cells (CHO-CAR, CHO-sialic acid, CHO-DSG and CHO-CD46 (K1, BC1, BC2, C1 and C2) were seeded as four replicates in 96-well tissue culture plates and infected with HAdV-20-42-42-Luc or HAd5-Luc at a concentration of 1 × 10^4^ VP/cell. Cell cultures were incubated 3 h at 37°C with 5 % CO_2_. Luciferase levels were measured at 48 to 72 h postinfection with a Victor X Multilabel plate reader (Perkin Elmer) by following the manufacturer’s instructions. Luciferase transgene expression was presented as luminescence relative light units per milligram of protein. All assays were performed with four sample replicates for each experimental condition.

### Surface plasmon resonance.

CM5 sensor chips and amine-coupling kits were purchased from GE Healthcare. All surface plasmon resonance (SPR) experiments were performed at 25°C in 20 mM HEPES, 150 mM NaCl, and 5 mM Ca^2+^ running buffer. Data were collected with a Biacore T200 instrument at a rate of 1 Hz. CD46 was coupled to the CM5 sensor chip by amine coupling reactions according to the manufacturer’s instructions, aiming for an immobilization density of 2,000 to 2,500 resonance units (RU). The surface of the upstream flow cell was used as a reference and was subjected to the same coupling reaction in the absence of protein. The hexon analyte (produced as described earlier) (17) was serially diluted in running buffer to prepare a 2-fold concentration series of 0.25 μM, 0.5 μM, and 1 μM and then injected over reference and experimental biosensor surfaces for 120 s at a flow rate of 30 μl/min. Blank samples containing only running buffer were also injected under the same conditions to allow for double referencing. After each cycle, the biosensor surface was regenerated with a 60-s pulse of 10 mM Tris-glycine (pH 1.5) at a flow rate of 30 μl/min.

### *In vivo* biodistribution.

All animal experiments were fully approved by University of Edinburgh Animal Procedures and Ethics Committee and performed under the UK Home Office license in accordance with the UK Home Office guidelines. Immunocompetent outbred MF1 male mice (Charles River Laboratories) aged 8 to 10 weeks were used for the 48 h postinjection biodistribution experiment. The animals were organized in six groups with five animals in each group, except the control (PBS) groups, which had 3 animals. In order to deplete circulating macrophages and more efficiently evaluate the transit of the virus at the whole organism level, 200 μl of clodronate liposomes (CL) was intravenously (i.v.) administered to corresponding groups at 48 h prior to virus administration.

Treatment groups were i.v. infected with a single dose (1 × 10^11^ VP diluted in 100 μl PBS) of HAdV-20-42-42-Luc or HAdV-5-Luc. Matched control groups were injected with 100 μl of PBS. At 48 h after virus delivery, luciferase activity was imaged with the method IVIS Spectrum (CaliperLife Science, UK). Prior to the imaging procedure, 0.5 ml luciferin was injected into the mice. Animals were maintained under inhalational anesthesia (AB-G). Luciferase activity detected ranged from low (shown in blue) to high (shown in red) levels. After the imaging was completed, animals were sacrificed and their organs (liver, heart, spleen, kidney, intestine, pancreas, and lungs) collected for the quantification of the vector genomes as described before (13).

For the 1 h postinjection time point, immunocompetent 8-week-old C57BL/6J male mice (Charles River Laboratories) were i.v. infected with a single dose (1 × 10^11^ VP diluted in 100 μl PBS) of HAdV-20-42-42-LacZ (*n* = 8) or HAdV-5-LacZ (*n* = 8). Matched control groups were injected with 100 μl of PBS (*n* = 4). At 1 h after virus delivery, animals were sacrificed and their organs (liver, heart, spleen, kidney, intestine, pancreas, and lungs) were collected for the quantification of the vector genomes.

### Serum cytokine analysis.

Immunocompetent 8-week-old C57BL/6J male mice (Charles River Laboratories) were i.v. infected with a single dose (1 × 10^11^ VP diluted in 100 μl PBS) of HAdV-20-42-42-LacZ (*n* = 8) or HAdV-5-LacZ (*n* = 8). Matched control groups were injected with 100 μl of PBS (*n* = 4). At 6 h after virus delivery, animals were sacrificed and blood was collected through cardiac puncture for serum isolation and subsequent cytokine analysis. Cytokine profiles were determined from 6 h postinjection sera using a V-PLEX mouse cytokine 19-plex kit (MSD) and a MESO SECTOR s 600 machine (Meso Scale Discovery).

### Mouse immunization study.

All animal experimentation was performed according to Dutch law and the guidelines on the protection of experimental animals published by the Council of the European Committee (45). Six-to-8-week-old specific-pathogen-free female BALB/c mice were purchased from Charles River and kept at the institutional animal facility under specified pathogen-free conditions. For prime immunization studies, mice were immunized intramuscularly with HAdV-20-42-42 or HAdV-26 vectors (1 × 10^9^ VP or 1 × 10^10^ VP per mouse) expressing Luc. Two weeks postimmunization, mice were sacrificed and the induction of Luc-specific IFN-γ-producing cells was measured by IFN-γ ELISPOT assay. In brief, mouse splenocytes were stimulated with 15-mer peptide pool spanning Luc (luc pool), medium (control negative), or phorbol myristate acetate (PMA; positive control).

Mice antisera against HAdV-20-42-42 and HAdV-26 were cross-tested against both vectors in an adenovirus neutralization assay. Starting from a 1:16 dilution, the sera were 2-fold serially diluted, as described elsewhere (26).

### Statistical analysis.

Statistical analysis was performed with GraphPad Prism software. One-way analysis of variance with the two-tailed Student's *t* test was used for statistical parameter comparison between different groups. The parameters of significance are indicated in each figure caption: *, *P < *0.05; **, *P < *0.005; ***, *P < *0.001; ns, not statistically significant (*P > *0.05). The presented *in vitro* results are averaged data from at least three different experiments with four experimental replicates per condition. The *in vivo* experiments were performed with a minimum of five animals per group. Errors bars represent the standard error of the mean (SEM). Statistical analyses were performed with SAS version 9.4 for Fig. 6. Noninferiority testing across doses was performed on log_10_-transformed data, with HAdV-26-Luc as a reference and a prespecified margin of 0.5 log_10_.

### Data availability.

The HAdV-20-42-42 genome sequence was submitted to the NCBI Nucleotide database with accession number MW694832.

## References

[1] Shirley JL, de Jong YP, Terhorst C, Herzog RW. 2020. Immune responses to viral gene therapy vectors. Mol Ther 28:709–722. 10.1016/j.ymthe.2020.01.001. 31968213 PMC7054714

[2] Singh S, Kumar R, Agrawal B. 2019. Adenoviral vector-based vaccines and gene therapies: current status and future prospects. Adenoviruses 10.5772/intechopen.79697.

[3] Sun Y, Lv X, Ding P, Wang L, Sun Y, Li S, Zhang H, Gao Z. 2019. Exploring the functions of polymers in adenovirus-mediated gene delivery: evading immune response and redirecting tropism. Acta Biomater 97:93–104. 10.1016/j.actbio.2019.06.059. 31386928

[4] Gao J, Zhang W, Ehrhardt A. 2020. Expanding the spectrum of adenoviral vectors for cancer therapy. Cancers 12:1139. 10.3390/cancers12051139. PMC7281331 32370135

[5] Ismail AM, Cui T, Dommaraju K, Singh G, Dehghan S, Seto J, Shrivastava S, Fedorova NB, Gupta N, Stockwell TB, Halpin R, Madupu R, Heim A, Kajon AE, Romanowski EG, Kowalski RP, Malathi J, Therese KL, Madhavan HN, Zhang Q, Ferreyra LJ, Jones MS, Rajaiya J, Dyer DW, Chodosh J, Seto D. 2018. Genomic analysis of a large set of currently—and historically—important human adenovirus pathogens. Emerg Microbes Infect 7:208–222. 10.1038/s41426-018-0200-4. 29410402 PMC5837155

[6] Robinson CM, Singh G, Lee JY, Dehghan S, Rajaiya J, Liu EB, Yousuf MA, Betensky RA, Jones MS, Dyer DW, Seto D, Chodosh J. 2013. Molecular evolution of human adenoviruses. Sci Rep 3:1812. 10.1038/srep01812. 23657240 PMC3648800

[7] Gonzalez G, Koyanagi KO, Aoki K, Watanabe H. 2015. Interregional coevolution analysis revealing functional and structural interrelatedness between different genomic regions in human mastadenovirus D. J Virol 89:6209–6217. 10.1128/JVI.00515-15. 25833048 PMC4474320

[8] Walsh MP, Chintakuntlawar A, Robinson CM, Madisch I, Harrach B, Hudson NR, Schnurr D, Heim A, Chodosh J, Seto D, Jones MS. 2009. Evidence of molecular evolution driven by recombination events influencing tropism in a novel human adenovirus that causes epidemic keratoconjunctivitis. PLoS One 4:e5635. 10.1371/journal.pone.0005635. 19492050 PMC2685984

[9] Kaján GL, Lipiec A, Bartha D, Allard A, Arnberg N. 2018. A multigene typing system for human adenoviruses reveals a new genotype in a collection of Swedish clinical isolates. PLoS One 13:e0209038. 10.1371/journal.pone.0209038. 30550551 PMC6294355

[10] Waddington SN, McVey JH, Bhella D, Parker AL, Barker K, Atoda H, Pink R, Buckley SM, Greig JA, Denby L, Custers J, Morita T, Francischetti IM, Monteiro RQ, Barouch DH, van Rooijen N, Napoli C, Havenga MJ, Nicklin SA, Baker AH. 2008. Adenovirus serotype 5 hexon mediates liver gene transfer. Cell 132:397–409. 10.1016/j.cell.2008.01.016. 18267072

[11] Qiu Q, Xu Z, Tian J, Moitra R, Gunti S, Notkins AL, Byrnes AP. 2015. Impact of natural IgM concentration on gene therapy with adenovirus type 5 vectors. J Virol 89:3412–3416. 10.1128/JVI.03217-14. 25552715 PMC4337529

[12] Alonso-Padilla J, Papp T, Kaján GL, Benkő M, Havenga M, Lemckert A, Harrach B, Baker AH. 2016. Development of novel adenoviral vectors to overcome challenges observed with HAdV-5-based constructs. Mol Ther 24:6–16. 10.1038/mt.2015.194. 26478249 PMC4754553

[13] Duffy MR, Alonso-Padilla J, John L, Chandra N, Khan S, Ballmann MZ, Lipiec A, Heemskerk E, Custers J, Arnberg N, Havenga M, Baker AH, Lemckert A. 2018. Generation and characterization of a novel candidate gene therapy and vaccination vector based on human species D adenovirus type 56. J Gen Virol 99:135–147. 10.1099/jgv.0.000978. 29154744

[14] Havenga M, Vogels R, Zuijdgeest D, Radosevic K, Mueller S, Sieuwerts M, Weichold F, Damen I, Kaspers J, Lemckert A, van Meerendonk M, van der Vlugt R, Holterman L, Hone D, Skeiky Y, Mintardjo R, Gillissen G, Barouch D, Sadoff J, Goudsmit J. 2006. Novel replication-incompetent adenoviral B-group vectors: high vector stability and yield in PER.C6 cells. J Gen Virol 87:2135–2143. 10.1099/vir.0.81956-0. 16847108

[15] Nevels M, Spruss T, Wolf H, Dobner T. 1999. The adenovirus E4orf6 protein contributes to malignant transformation by antagonizing E1A-induced accumulation of the tumor suppressor protein p53. Oncogene 18:9–17. 10.1038/sj.onc.1202284. 9926915

[16] Arnberg N. 2012. Adenovirus receptors: implications for targeting of viral vectors. Trends Pharmacol Sci 33:442–448. 10.1016/j.tips.2012.04.005. 22621975

[17] Persson BD, John L, Rafie K, Strebl M, Frängsmyr L, Ballmann MZ, Mindler K, Havenga M, Lemckert A, Stehle T. 2021. Human species D adenovirus hexon capsid protein mediates cell entry through a direct interaction with CD46. Proc Natl Acad Sci USA 118. 10.1073/pnas.2020732118. PMC7826407 33384338

[18] Shayakhmetov DM, Gaggar A, Ni S, Li ZY, Lieber A. 2005. Adenovirus binding to blood factors results in liver cell infection and hepatotoxicity. J Virol 79:7478–7491. 10.1128/JVI.79.12.7478-7491.2005. 15919903 PMC1143681

[19] Parker AL, Waddington SN, Nicol CG, Shayakhmetov DM, Buckley SM, Denby L, Kemball-Cook G, Ni S, Lieber A, McVey JH, Nicklin SA, Baker AH. 2006. Multiple vitamin K-dependent coagulation zymogens promote adenovirus-mediated gene delivery to hepatocytes. Blood 108:2554–2561. 10.1182/blood-2006-04-008532. 16788098

[20] Coughlan L, Bradshaw AC, Parker AL, Robinson H, White K, Custers J, Goudsmit J, Van Roijen N, Barouch DH, Nicklin SA, Baker AH. 2012. Ad5:Ad48 hexon hypervariable region substitutions lead to toxicity and increased inflammatory responses following intravenous delivery. Mol Ther 20:2268–2281. 10.1038/mt.2012.162. 22929662 PMC3514487

[21] Bradshaw AC, Coughlan L, Miller AM, Alba R, van Rooijen N, Nicklin SA, Baker AH. 2012. Biodistribution and inflammatory profiles of novel penton and hexon double-mutant serotype 5 adenoviruses. J Control Release 164:394–402. 10.1016/j.jconrel.2012.05.025. 22626939 PMC3520007

[22] Milligan ID, Gibani MM, Sewell R, Clutterbuck EA, Campbell D, Plested E, Nuthall E, Voysey M, Silva-Reyes L, McElrath MJ, De Rosa SC, Frahm N, Cohen KW, Shukarev G, Orzabal N, van Duijnhoven W, Truyers C, Bachmayer N, Splinter D, Samy N, Pau MG, Schuitemaker H, Luhn K, Callendret B, Van Hoof J, Douoguih M, Ewer K, Angus B, Pollard AJ, Snape MD. 2016. Safety and immunogenicity of novel adenovirus type 26- and modified vaccinia Ankara-vectored Ebola vaccines: a randomized clinical trial. JAMA 315:1610–1623. 10.1001/jama.2016.4218. 27092831

[23] Baden LR, Karita E, Mutua G, Bekker LG, Gray G, Page-Shipp L, Walsh SR, Nyombayire J, Anzala O, Roux S, Laher F, Innes C, Seaman MS, Cohen YZ, Peter L, Frahm N, McElrath MJ, Hayes P, Swann E, Grunenberg N, Grazia-Pau M, Weijtens M, Sadoff J, Dally L, Lombardo A, Gilmour J, Cox J, Dolin R, Fast P, Barouch DH, Laufer DS, B003-IPCAVD004-HVTN091 Study Group. 2016. Assessment of the safety and immunogenicity of 2 novel vaccine platforms for HIV-1 prevention: a randomized trial. Ann Intern Med 164:313–322. 10.7326/M15-0880. 26833336 PMC5034222

[24] Mercado NB, Zahn R, Wegmann F, Loos C, Chandrashekar A, Yu J, Liu J, Peter L, McMahan K, Tostanoski LH, He X, Martinez DR, Rutten L, Bos R, van Manen D, Vellinga J, Custers J, Langedijk JP, Kwaks T, Bakkers MJG, Zuijdgeest D, Rosendahl Huber SK, Atyeo C, Fischinger S, Burke JS, Feldman J, Hauser BM, Caradonna TM, Bondzie EA, Dagotto G, Gebre MS, Hoffman E, Jacob-Dolan C, Kirilova M, Li Z, Lin Z, Mahrokhian SH, Maxfield LF, Nampanya F, Nityanandam R, Nkolola JP, Patel S, Ventura JD, Verrington K, Wan H, Pessaint L, Van Ry A, Blade K, Strasbaugh A, Cabus M, et al. 2020. Single-shot Ad26 vaccine protects against SARS-CoV-2 in rhesus macaques. Nature 586:583–586. 10.1038/s41586-020-2607-z. 32731257 PMC7581548

[25] Poland GA, Ovsyannikova IG, Crooke SN, Kennedy RB. 2020. SARS-CoV-2 vaccine development: current status. Mayo Clin Proc 95:2172–2188. 10.1016/j.mayocp.2020.07.021. 33012348 PMC7392072

[26] Sprangers MC, Lakhai W, Koudstaal W, Verhoeven M, Koel BF, Vogels R, Goudsmit J, Havenga MJ, Kostense S. 2003. Quantifying adenovirus-neutralizing antibodies by luciferase transgene detection: addressing preexisting immunity to vaccine and gene therapy vectors. J Clin Microbiol 41:5046–5052. 10.1128/JCM.41.11.5046-5052.2003. 14605137 PMC262545

[27] Sayedahmed EE, Elkashif A, Alhashimi M, Sambhara S, Mittal SK. 2020. Adenoviral vector-based vaccine platforms for developing the next generation of influenza vaccines. Vaccines 8:574. 10.3390/vaccines8040574. PMC7712206 33019589

[28] Abbink P, Lemckert AA, Ewald BA, Lynch DM, Denholtz M, Smits S, Holterman L, Damen I, Vogels R, Thorner AR, O'Brien KL, Carville A, Mansfield KG, Goudsmit J, Havenga MJ, Barouch DH. 2007. Comparative seroprevalence and immunogenicity of six rare serotype recombinant adenovirus vaccine vectors from subgroups B and D. J Virol 81:4654–4663. 10.1128/JVI.02696-06. 17329340 PMC1900173

[29] Lemckert AAC, Grimbergen J, Smits S, Hartkoorn E, Holterman L, Berkhout B, Barouch DH, Vogels R, Quax P, Goudsmit J, Havenga MJE. 2006. Generation of a novel replication-incompetent adenoviral vector derived from human adenovirus type 49: manufacture on PER.C6 cells, tropism and immunogenicity. J Gen Virol 87:2891–2899. 10.1099/vir.0.82079-0. 16963747

[30] Baden LR, Walsh SR, Seaman MS, Tucker RP, Krause KH, Patel A, Johnson JA, Kleinjan J, Yanosick KE, Perry J, Zablowsky E, Abbink P, Peter L, Iampietro MJ, Cheung A, Pau MG, Weijtens M, Goudsmit J, Swann E, Wolff M, Loblein H, Dolin R, Barouch DH. 2013. First-in-human evaluation of the safety and immunogenicity of a recombinant adenovirus serotype 26 HIV-1 Env vaccine (IPCAVD 001). J Infect Dis 207:240–247. 10.1093/infdis/jis670. 23125444 PMC3532831

[31] Teigler JE, Iampietro MJ, Barouch DH. 2012. Vaccination with adenovirus serotypes 35, 26, and 48 elicits higher levels of innate cytokine responses than adenovirus serotype 5 in rhesus monkeys. J Virol 86:9590–9598. 10.1128/JVI.00740-12. 22787208 PMC3446581

[32] Kajon AE, Erdman DD. 2007. Assessment of genetic variability among subspecies B1 human adenoviruses for molecular epidemiology studies. Methods Mol Med 131:335–355. 10.1007/978-1-59745-277-9_23. 17656793

[33] Chevreux B, Wetter T, Suhai S. 1999. Genome sequence assembly using trace signals and additional sequence information. German Conf Bioinf 99:1.

[34] Kearse M, Moir R, Wilson A, Stones-Havas S, Cheung M, Sturrock S, Buxton S, Cooper A, Markowitz S, Duran C, Thierer T, Ashton B, Meintjes P, Drummond A. 2012. Geneious Basic: an integrated and extendable desktop software platform for the organization and analysis of sequence data. Bioinformatics 28:1647–1649. 10.1093/bioinformatics/bts199. 22543367 PMC3371832

[35] Yuan X, Qu Z, Wu X, Wang Y, Liu L, Wei F, Gao H, Shang L, Zhang H, Cui H, Zhao Y, Wu N, Tang Y, Qin L. 2009. Molecular modeling and epitopes mapping of human adenovirus type 3 hexon protein. Vaccine 27:5103–5110. 10.1016/j.vaccine.2009.06.041. 19573641

[36] Katoh K, Standley DM. 2013. MAFFT multiple sequence alignment software version 7: improvements in performance and usability. Mol Biol Evol 30:772–780. 10.1093/molbev/mst010. 23329690 PMC3603318

[37] Kozlov AM, Darriba D, Flouri T, Morel B, Stamatakis A. 2019. RAxML-NG: a fast, scalable and user-friendly tool for maximum likelihood phylogenetic inference. Bioinformatics 35:4453–4455. 10.1093/bioinformatics/btz305. 31070718 PMC6821337

[38] Capella-Gutierrez S, Silla-Martinez JM, Gabaldon T. 2009. trimAl: a tool for automated alignment trimming in large-scale phylogenetic analyses. Bioinformatics 25:1972–1973. 10.1093/bioinformatics/btp348. 19505945 PMC2712344

[39] Darriba D, Posada D, Kozlov AM, Stamatakis A, Morel B, Flouri T. 2020. ModelTest-NG: a new and scalable tool for the selection of DNA and protein evolutionary models. Mol Biol Evol 37:291–294. 10.1093/molbev/msz189. 31432070 PMC6984357

[40] Kumar S, Stecher G, Tamura K. 2016. MEGA7: molecular evolutionary genetics analysis version 7.0 for bigger datasets. Mol Biol Evol 33:1870–1874. 10.1093/molbev/msw054. 27004904 PMC8210823

[41] Lole KS, Bollinger RC, Paranjape RS, Gadkari D, Kulkarni SS, Novak NG, Ingersoll R, Sheppard HW, Ray SC. 1999. Full-length human immunodeficiency virus type 1 genomes from subtype C-infected seroconverters in India, with evidence of intersubtype recombination. J Virol 73:152–160. 10.1128/JVI.73.1.152-160.1999. 9847317 PMC103818

[42] Bergelson JM, Cunningham JA, Droguett G, Kurt-Jones EA, Krithivas A, Hong JS, Horwitz MS, Crowell RL, Finberg RW. 1997. Isolation of a common receptor for coxsackie B viruses and adenoviruses 2 and 5. Science 275:1320–1323. 10.1126/science.275.5304.1320. 9036860

[43] Liszewski MK, Atkinson JP. 1996. Membrane cofactor protein (MCP; CD46). Isoforms differ in protection against the classical pathway of complement. J Immunol 156:4415–4421. 8666815

[44] Maizel JV, Jr, White DO, Scharff MD. 1968. The polypeptides of adenovirus. I. Evidence for multiple protein components in the virion and a comparison of types 2, 7A, and 12. Virology 36:115–125. 10.1016/0042-6822(68)90121-9. 5669982

[45] Legislation for the protection of animals used for scientific purposes. 2019. Directive 2010/63/EU as amended by Regulation (EU) 2019/1010. https://ec.europa.eu/environment/chemicals/lab_animals/legislation_en.htm.

